# Clinical applications of exosomes in cosmetic dermatology

**DOI:** 10.1002/ski2.348

**Published:** 2024-02-13

**Authors:** Ge Bai, Thu Minh Truong, Gaurav N. Pathak, Lora Benoit, Babar Rao

**Affiliations:** ^1^ Department of Dermatology Rutgers Robert Wood Johnson Medical School Piscataway New Jersey USA; ^2^ Department of Dermatology Rutgers New Jersey Medical School Newark New Jersey USA; ^3^ IEH Laboratories and Consulting Group Lake Forest Park Washington USA; ^4^ Department of Dermatology Weill School of Medicine New York New Jersey USA

## Abstract

**Introduction:**

Exosomes are extracellular vesicles that transport bioactive substances during normal and abnormal cellular physiological processes. The unique properties of exosomes can be exploited for use as biomarkers and targeted drug delivery vehicles, and are, for this reason, gaining increasing attention in the field of dermatology. This review aims to synthesise the existing evidence supporting exosomes in regenerative and cosmetic dermatology.

**Method:**

A comprehensive PubMed search for the period of 2010–2023 was performed using the MeSH terms "exosome" and "skin.” The initial search yielded 246 studies, which were then refined to 178 studies following title and abstract screening. Studies were confined to human or animal studies published in English that evaluated the use of exosomes in medical/cosmetic dermatology. A subsequent full‐text review based on these criteria yielded 34 studies, which were then reviewed.

**Results:**

Exosomes can be derived from a variety of biological sources and show potential application in wound healing, scar prophylaxis, photodamage prevention, skin regeneration, improved grafting success, hair loss mitigation, and as biomarkers and drug carriers.

**Conclusion:**

Exosomes are gaining traction in regenerative and cosmetic dermatology. However, their widespread clinical application is hindered by cost, a complex isolation process, lack of uniform protocols, limited assessment of infective potential, and a paucity of clinical evidence. Further research in this area is needed, especially by way of clinical studies evaluating the efficacy of exosome‐based treatments on human skin.

## Graphical abstract

Exosomes can be derived from a variety of biological sources and show potential application in wound healing, scar prophylaxis, photodamage prevention, skin regeneration, improved grafting success, hair loss mitigation, and as biomarkers and drug carriers. However, their widespread clinical application is hindered by cost, a complex isolation process, lack of uniform experimental protocols, and limited assessment of infective potential hinder their widespread clinical application.

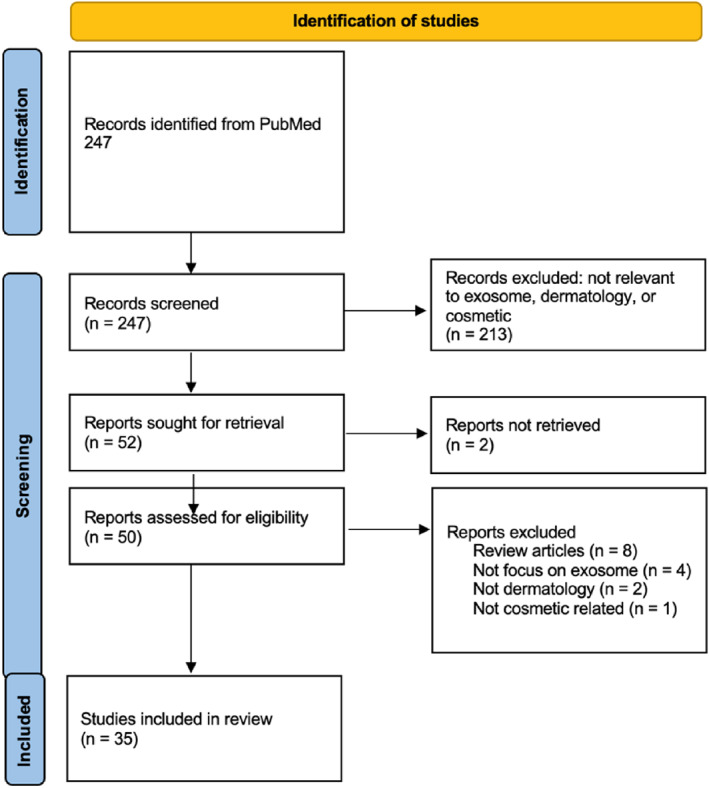

**What's already known about this topic?**

- Exosomes are extracellular vesicles that transport bioactive substances during normal and abnormal cellular physiological processes. The unique properties of exosomes can be exploited for use as biomarkers and targeted drug delivery vehicles, and are, for this reason, gaining increasing attention in the field of dermatology.

**What does this study add?**

- This review aims to synthesize the existing evidence supporting exosomes in regenerative and cosmetic dermatology.

## 1 INTRODUCTION

Exosomes are nano‐sized EV ranging in size from 40 to 160 nm that are produced from within the endosomal compartment of most eukaryotic cells. Exosomes transport various biomolecules including nucleic acids, lipids, proteins, and metabolites required for cellular physiology as well as signalling molecules that facilitate intercellular communication.^1^ Exosomes encapsulate biomolecules within membranes that are studded with transmembrane proteins and enriched with cholesterol, sphingomyelin, and lipid rafts. These features enable exosomes to delivery internal cargo to neighbouring cells through receptor‐mediated endocytosis,^2^ a property that can be exploited in targeted cell‐free delivery systems. Related to cosmetic dermatology, exosomes have significant regenerative and cosmetic potential on account of their ability to stimulate neoangiogenesis, reverse fibroblast senescence and keratinocyte atrophy, promote synthesis of extracellular matrix components, and regulate oxidative stress and inflammation.^3^, ^4^

While studies to date have amassed substantial evidence supporting the utility of exosomes as drug delivery vehicles, their practical implementation in medical settings lags behind. To bridge this gap and advance our understanding of exosomes in dermatological treatments, we conducted a comprehensive narrative literature review. Our review emphasises key aspects such as potential clinical applications, biological sources, derivation methods, treatment outcomes, and safety considerations. By consolidating the current body of literature in this field, our aim is to facilitate a broader adoption of exosomes in regenerative and cosmetic dermatology.

## 2 METHODS

**FIGURE 1:**
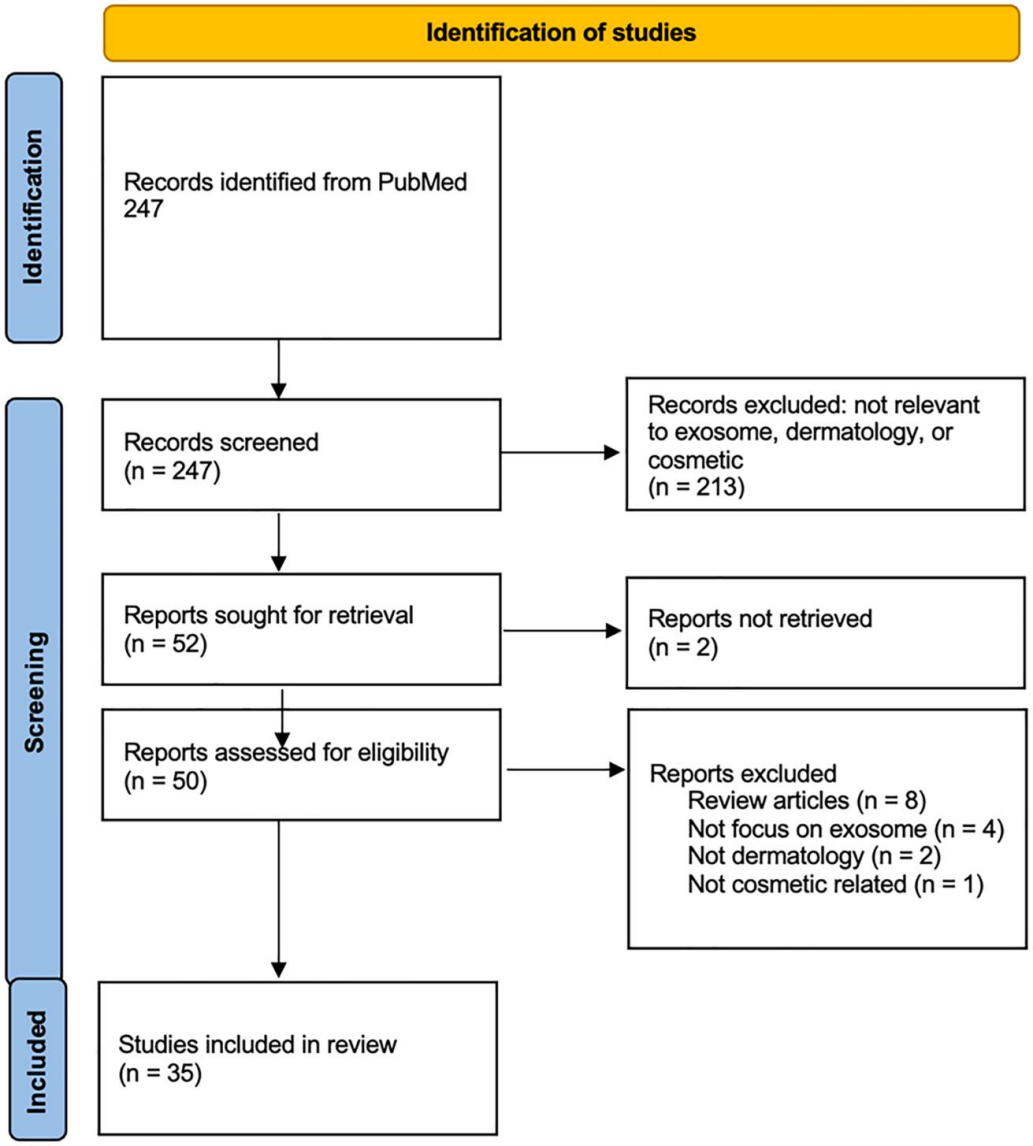
Prisma flow diagram.

A narrative literature review was performed by interrogating the PubMed database search for the period of 2010–2023 with the MeSH (Medical Subject Headings) terms "exosome" and "skin." The initial search yielded a total of 247 results. Title and abstract screening on the basis relevancy to exosomes in dermatology reduced this number to 178 reports. A full‐text review was then conducted to identify animal or human studies that examined the use of exosomes in cosmetic dermatologic conditions or applications. Six studies written in languages other than English were excluded. A total of 35 articles were retrieved using this scheme. See Figure 1. Flow Chart of Study Selection. Of these, four clinical studies were identified and summarised, see Table 1. The remaining non‐clinical studies were categorised into parameters of interest and reviewed accordingly.

**TABLE 1:** Summary of all Clinical Studies involving Human Subjects.

Study (ref)	Study design	N	Treatment	Tools	Outcome
Park, 2023^11^	Prospective, randomized, split‐face, comparative study	28	Human ASCE‐containing solution (HACS)	Microneedling device, PRIMOS premium, Cutometer MPA 580, Corneometer CM 825, and Mark‐Vu	HACS combined with micro‐needling treated arm scored higher than the control arm (micro‐needling only) in the Global Aesthetic Improvement Scale score at week 6 (*p* = 0.023) and follow up visit at week 12 (*p* = 0.005). At week 12, skin wrinkle reduction in the HACS treated side was 13.4% while control was 7.1% (*p* = 0.007); skin elasticity increased 11.3% in the HACS arm while control decreased by 3.3% (*p* = 0.002); skin hydration increased 6.5% (control is 4.5%, *p* = 0.037); melanin decreased by 9.9% (control decreased by 1%, *p* = 0.044); histology slides showed more collagen and elastic fibre deposition compared to control side.
Proffer, 2022^21^	Prospective, single‐arm, non‐randomized, longitudinal study	56	Human platelet‐derived exosomes	VISIA‐CR imaging, measuring change from baseline	At 6 weeks, the skin health score improved by 224.2 ± 112.8 skin health score units (*p* ≤ 0.0001) from baseline. Reduction in redness (*p* = 0.005), wrinkles (*p* = 0.0023), melanin production (*p* ≤ 0.0001), enhanced luminosity and colour evenness (*p* ≤ 0.001).
Chernoff, 2023^23^	Prospective, four‐arm, non‐randomized, longitudinal study	40	Placental derived mesenchymal stem cell‐exosomes	Quantificare 3D photo documentation and skin analysis	Patients treated with topical emulsion of exosomes experienced betterment in wrinkles, pores, skin evenness, vascularity, and decreased oiliness and pigment after 30 days following treatment.
Jo, 2022^24^	Prospective, single‐arm, non‐randomized, longitudinal study	16	*Lactobacillus plantarum*‐derived exosomes (LpEV group)	MARK Vu and F‐ray equipment	Patients in the LpEV group had a reduction in eye wrinkles by 8.9% and 15.89% at 2 and 4 weeks, with no change in the placebo group; an increase in skin elasticity by 14.76% and 27.07% at 2 and 4 weeks, respectively; increase in water content by 10.79% and 21.40% at 2 and 4 weeks; and image analysis showed higher skin density growth in the LpEV group (39.30%) compared to placebo (15.19%), with LpEV‐associated density improvements of 3.87% and 8.7% at 2 and 4 weeks.

*Note*: PRIMOS Premium for facial wrinkle measurement. Cutometer MPA 580 for skin elasticity measurement. Corneometer CM 825 for skin hydration measurement. MARK Vu is a LED skin analyser for a variety of skin conditions. F‐ray is to measure skin contours. Quantificare is for 3D photo documentation and skin analysis.

Abbreviations: ASCE, Adipose tissue stem cell‐derived exosomes; CaHA, calcium hydroxyapatite; CR, complexion report; LED, light emitting diode; LePV, L. plantarum extracellular vesicle; VISIA, system name.

## 3 RESULTS

### 3.1 The role of exosomes in reducing photo‐oxidative stress

Ageing skin is particularly vulnerable to the detrimental effects of oxidative stress and inflammation due to fibroblast senescence, which occurs because of ageing. A study by Hu et al. showed that senescent fibroblast functionality could be restored using a needle‐free jet injector system to deliver 3D spheroid‐derived exosomes prepared from human dermal fibroblast (HDFs) (HDF‐XOs). Using a combination of both in vitro studies and a nude mouse model of photo‐ageing, increased collagen production by dermal fibroblasts was observed as well as thickening of the dermal matrix.^5^ These findings suggest that transdermal delivery of HDF‐XOs offers a promising treatment option for combating age‐related changes in the skin.

Exposure of skin to UV radiation generates hydrogen peroxide (H_2_O_2_) and various other reactive oxygen species (ROS) which contributes to the development of skin pathologies and accelerates the ageing process. A study by Wu et al. revealed that exosomes derived from human umbilical cord mesenchymal stem cells (hucMSC‐ex) could mitigate UV‐induced photodamaged skin in rats by reducing ROS production and inflammation within the tissue.^6^ A mechanism of action was elucidated involving upregulation of the SIRT1/Nrf2 pathway in keratinocytes, thereby protecting against oxidative damage and extending photoprotective effects by increasing autophagy while decreasing apoptosis within the skin.

Similarly, exosomes derived from adipose tissue express intact antioxidant signalling capacity. In this regard, Gao et al. showed that adipose‐derived stem cell exosomes (ADSC‐exosomes or ADSCs‐Exo) enhanced cell viability, suppressed ROS induced‐DNA damage, and inhibited the expression of matrix metalloproteinases (MMPs) in UVB‐irradiated HDFs in vitro.^7^ ADSC‐exo also promoted procollagen synthesis, in a manner that was dependent on mitogen activated protein kinases (MAPK)/AP‐1 and TGF‐β/Smad signalling pathways. Furthermore, like umbilical cord stem cell exosomes, ADSCs‐Exo were shown to upregulate the SIRT1/Nrf2 pathway.

ADSC‐exosomes have also been shown to promote rejuvenation and repair of photodamaged skin. In this regard, Liang et al. demonstrated using a rat model of photo‐ageing in which subcutaneously injected ADSC‐exosomes promoted thickening of the epidermis and stratum corneum and reduced aberrant cellular proliferation.^8^

### 3.2 Regenerative capacities of exosomes: Hair, skin and more

Ageing and prolonged solar exposure can significantly affect hair growth and skin pigmentation, resulting in hair loss and dyspigmentation. Exosomes participate in skin homoeostasis by facilitating intercellular transport of microRNA. A study by Shen et al. showed that UV‐B‐induced alterations in exosomal miRNA derived from primary human melanocytes could influence cell cycle control, DNA repair, autophagy, and oncogenesis.^9^ Likewise, keratinocyte‐released exosomes have been shown to modulate melanocyte pigmentation through miRNA transfer, thereby impacting skin pigmentation.^10^ A recent split‐face clinical trial undertaken by Park et al. demonstrated that human ADSC‐exosomes combined with micro‐needling improved skin elasticity and hydration, while reducing pigmentation and the appearance of wrinkles (see Table 1).^11^ Similarly, Han et al. reported that bovine colostrum‐derived exosomes prevented excessive melanin formation and promoted photo damage repair through increased collagen production in vitro.^12^

Hair growth has been achieved using exosomes derived from the fungus *Polyporus linteus*, dermal fibroblasts, and ADSCs.^13^, ^14^, ^15^, ^16^, ^17^, ^18^ In addition, animal‐derived exosomes have proven to be effective as well. In this line, Kim et al. showed the potential of bovine colostrum‐derived exosomes in activating the Wnt/β‐catenin pathway in hair dermal papillary cells in vitro as well as in mice, stimulating dermal papilla cell proliferation and follicle regeneration.^19^

Hair growth can be permanently affected by chemotoxic medication and radiation. A study by Ariyoshi et al. showed that exosomes derived from mouse buccal tissue can promote hair growth and prevent hair loss after radiation treatment by inducing faster DNA repair in neonatal mice. Normal hair growth was observed 2 weeks following radiation treatment. The study also showed increased kinetics of DNA repair in the exosome‐treated skin compared to the untreated irradiated control area.^20^

In addition to hair restoration, the use of exosomes in skin rejuvenation is another area of active research. Proffer et al. demonstrated the efficacy of platelet‐derived exosomes when applied topically in human subjects, which showed improvement in erythema, pigmentation, luminosity, and wrinkles (see Table 1).^21^, ^22^ Shieh et al. used mesenchymal stem cell‐derived exosome after bio‐pulsation, and showed enhanced bioactivity in promoting cell proliferation, wound healing, collagen synthesis, and anti‐inflammatory effects in skin fibroblast and hair follicle cells.^21^ A connection between skin youthfulness and the constituents of its microbiome was explored in a clinical study by Chernoff, which showed high patient satisfaction with the use of exosome bio‐stimulatory dermal Infusions and improved skin tone and texture, along with reduced wrinkles, pores, pigment, and oiliness (see Table 1).^23^

As with human and animal‐derived sources, bacterial and plant‐derived sources can be used to generate exosomes as well. Jo et al. investigated the effects of exosomes derived from *Lactobacillus plantarum*, a bacterial constituent of the normal skin microbiome, on skin ageing of human subjects. Their research demonstrated the potential of these exosomes in reducing wrinkle formation, boosting skin moisture content, and mitigating pigmentation disorders through the regulation of ECM‐related genes (see Table 1).^24^ A separate study revealed that exosome‐like nanoparticles derived from beetroot extract have promising antioxidant and proangiogenic properties. Notably, they also boosted the activity of the hyaluronan synthase enzyme, which contributes to skin rejuvenation through enhanced moisture retention and collagen synthesis.^25^

### 3.3 Chronic wound repair: Expediting physiologic regeneration

Given their regenerative properties, an array of different human stem cell sources has been used to generate exosomes for the repair of chronic wounds. Zhang et al. demonstrated that ADSC‐ exosomes could augment wound healing by stimulating fibroblast activity and collagen deposition through PI3K/Akt signalling in vitro.^26^ In addition, exosome activity can be enhanced by external factors. For example, Yang et al. demonstrated that when human umbilical cord mesenchymal stem cells in mice were exposed to monochromatic blue light, there was an increase in miRNA content, specifically miR‐135b‐5p and miR‐499a‐3p, suggesting a novel approach to wound repair.^27^ The choice of delivery method may also enhance the clinical efficacy of exosomes. Nooshabadi et al. combined exosomes with a chitosan‐glycerol hydrogel in mice, noting enhanced wound healing marked by accelerated wound closure changes within 7 days of treatment and complete wound closure by day 14.^27^, ^28^ Furthering work on hydrogels, Li et al. engineered a hydrogel with dual capacity to load and release human umbilical cord derived mesenchymal stem cell exosomes in a rat model, allowing for a more controlled and stable delivery.^29^

### 3.4 Wound healing: Preventing pathologic scarring

A number of studies have evaluated the use of exosomes derived from various biological sources in the prevention or correction of pathologic scarring during the wound repair process. Of note, exosomes derived from post‐burn hypertrophic scar tissue exhibit an altered constitution that contributes to fibroblast proliferation and dermal thickening, with decreased interleukin‐1 alpha and increased platelet‐derived growth factor, which ultimately leads to scar hypertrophy.^30^ This finding infers that replenishment of signalling factors by exogenous exosomes can be potentially used for scar prophylaxis. In this regard, epidermal stem cell‐derived exosomes have been shown to suppress myoblast differentiation via inhibition of TGF‐beta/Smad signalling using a rat model.^31^ Furthermore, the application of human bone marrow‐derived MSC exosomes in mice has been shown to reduce local inflammation, over‐production of collagen, likewise through inhibition of the TGF‐beta/Smad pathway.^32^ Using mice models, Wang et. al and Zhou et. al both showed that ASC‐derived exosomes could influence the ratios of collagen type 1 and collagen type 2 synthesis, myofibroblast differentiation, and extracellular signal regulated kinase/MAPK signalling, which is integral in tissue remodelling.^33^, ^34^

The constituents of exosomes have also been investigated in the context of scar prevention, with emphasis on microRNA, which can influence diverse downstream targets that affect the expression of key gene products. Exosomes derived from human adipose stem cells could impede scar formation in mice when modified with microRNA 29a. MicroRNA 29a acts by inhibiting the TGF‐beta2/Smad3 signalling pathway, subsequently reducing collagen deposition and pathological scar formation.^35^ Similarly, Fang et al found that MSC‐exosomes containing various microRNAs suppressed myofibroblast formation via the inhibition of smooth muscle actin and collagen deposition, ultimately contributing to scar reduction in skin defect mice model.^36^ Another study demonstrated exosomes sourced from young fibroblasts enhanced wound healing in aged mice, an effect attributed to a microRNA 125b. As a regulatory molecule, miR‐125b targets Sir7 and activates the TGF‐beta1 pathway, thereby promoting wound healing.^37^ Another significant microRNA cargo in MSC‐exosomes, miR‐138‐5p, was shown to downregulate Sirt1 expression, attenuating pathological scarring in a skin defect mice model.^38^ For this reason, exosomal microRNAs have potential applications in personalised medicine.

### 3.5 Current clinical trials

A total of four prospective clinical trials were identified by this study, all of which focused on skin rejuvenation. Park et al. conducted a comparative study on 28 individuals, finding that application of human adipose stem cell‐derived exosomes containing solution (HACS) in conjunction with microneedling significantly improved skin aesthetics over microneedling alone following 12 weeks of treatment, with reported improvements in collagen content, wrinkle reduction, elasticity, hydration, and dyspigmentation.^11^ Proffer et al. conducted a 6‐week clinical trial involving 56 participants using a topically applied serum containing human platelet‐derived exosomes.^21^ Results showed significantly improved skin health and reduced redness, wrinkles, and melanin production. Chernoff et al. reported in a study involving 40 subjects. Here, topical application of an emulsion containing a mixture of placental‐derived MSC‐exosomes, botulinum toxin, and hyaluronic acid, followed by cavitating ultrasound and then by high‐intensity light emitting diode therapy improved skin texture, evenness, pore size, and decreased oiliness and dyspigmentation after 30 days.^23^ Lastly, Jo et al. found that *Lactobacillus plantarum*‐derived exosomes applied to the skin achieved a reduction in eye wrinkles and improvements in skin elasticity, hydration, and density in 16 volunteers after 4‐week of treatment.^24^

Currently, the market offers a variety of exosome‐containing products for treating facial skin and hair. However, as exosomes have not yet received food and drug administration approval, there are no established standards for their sourcing and preparation. Moreover, the exosome sources in these products are vaguely defined. Although some are marketed as daily home treatments, many of these products are designed to be used alongside minimally invasive procedures, such as laser treatments and micro‐needling. Despite their growing popularity, the paucity of clinical evidence does not yet warrant their widespread application in cosmetic dermatology.

## 4 CONCLUSION

In summary, our review highlights the extensive exploration of stem cell‐derived exosomes for chronic wound healing and scar reduction, revealing promising outcomes. Various stem cell sources, including adipose‐derived stem cells and mesenchymal stem cells, have demonstrated efficacy in wound healing and scar reduction. Moreover, interventions like blue light exposure and hydrogel integration appear to augment their therapeutic potential. Significantly, the role of microRNAs within exosome cargo has emerged as a critical determinant of their therapeutic effects, targeting specific pathways and molecules essential for wound healing and scar mitigation. Furthermore, exosomes show promise in the treatment areas of skin and hair regeneration. These findings collectively underscore the clinical potential of exosomes in regenerative medicine, laying the foundation for future translational applications. Additionally, exosomes exhibit promise in treating various skin disorders owing to their intercellular role and drug delivery capabilities, as well as their utility as biomarkers for conditions such as melanoma and fibrosis. However, the current clinical trials investigating the cosmetic application of exosome are limited, usually conduct by clinicians on a handful of volunteer patients. Therefore, future advancement of exosomes in cosmetic dermatology necessitates the harmonisation of study protocols and rigorous testing in clinical trials to establish appropriate usage methods and doses.

## AUTHOR CONTRIBUTIONS

**Ge Bai**: Data curation (equal); Investigation (equal); Visualisation (lead); Writing – original draft (lead); Writing – review & editing (lead). **Thu Minh Truong**: Project administration (equal); Resources (equal); Writing – original draft (supporting); Writing – review & editing (equal). **Gaurav N. Pathak**: Data curation (equal); Writing – original draft (supporting); Writing – review & editing (supporting). **Lora Benoit**: Supervision (supporting); Writing – review & editing (equal). **Barbar Rao**: Conceptualisation (lead); Supervision (lead); Validation (lead); Writing – review & editing (supporting).

## CONFLICT OF INTEREST STATEMENT

Dr. Rao is a speaker for Incyte. All other authors have no disclosures.

## ETHICS STATEMENT

Not applicable.

## Data Availability

The data that support the findings of this study are available from the corresponding author upon reasonable request.
